# Effects and Mechanisms of Hooked-End Steel Fibers and Carbon Nanotubes on the Shrinkage of Lithium Slag-Based Geopolymers: Role of Fiber-Confined Zones

**DOI:** 10.3390/ma19173656

**Published:** 2026-08-28

**Authors:** Cai Wu, Zhuo Luo, Xueping Zhou, Daopei Zhu

**Affiliations:** 1 School of Civil Engineering, Hubei Engineering University, Xiaogan 432000, China; 2 School of Civil Engineering and Surveying & Mapping Engineering (Nanchang), Jiangxi University of Science and Technology, Ganzhou 341000, China

**Keywords:** geopolymer, shrinkage, hooked-end steel fibers, carbon nanotubes, lithium slag, hybrid fibers, fiber–matrix interface, fiber-confined zone

## Abstract

Lithium slag-based geopolymer (LSG) provides a promising route for the high-value utilization of lithium slag; however, its pronounced shrinkage deformation continues to restrict engineering applications. To clarify the multi-scale mechanism by which fibers regulate LSG shrinkage, this study investigated the effects of single and hybrid additions of carbon nanotubes (CNTs) and hooked-end steel fibers (HSFs). The 90 d drying shrinkage test quantified shrinkage-reduction efficiency for mono- and hybrid-fiber systems. MIP was used for porosity and pore size distribution analysis; SEM examined fiber dispersion, interfacial bonding, and CNT-HSF synergy. At 90 d, 0.15% CNTs and 1.5% HSFs reduced shrinkage by 5.7% and 26.8%, respectively, whereas the C0.15-H1.5 hybrid mixture achieved a maximum reduction of 31.33% relative to the control. HSF addition increased porosity from 15.5% to 23.1% and the average pore diameter from 23.36 to 50.82 nm. Macroscopic shrinkage was reduced because interfacial friction and hooked-end anchorage provided mechanical restraint. In addition, based on fiber pull-out behavior and a simplified interfacial bond-slip model, the effective confinement radius at the fiber–matrix interface was analyzed, and the concept of fiber-confined zones was proposed. Results show that CNTs refine pore structure and bridge microcracks, whereas HSFs provide mechanical restraint through interfacial friction and anchorage. The hybrid CNT-HSF system achieved a maximum drying shrinkage reduction of 31.33%, higher than the corresponding theoretical additive value. The spatial overlap of fiber-confined zones is identified as the key mechanism for forming a weakly rigid framework that suppresses macroscopic shrinkage.

## 1. Introduction

The global transition toward green and low-carbon development, together with the rapid expansion of the new energy industry, has sharply increased the demand for lithium, a key metal for energy storage technologies [1,2]. Although the large-scale exploitation of lithium resources and the industrial production of lithium salts have supported the growth of the lithium battery sector, they have also generated substantial quantities of lithium slag [3,4].

Geopolymers are a class of inorganic binders within the broader family of alkali-activated materials (AAMs), generally produced through the reaction of amorphous or semicrystalline aluminosilicate precursors with alkaline activators [5,6,7] Compared with ordinary Portland cement (OPC), geopolymer production avoids high-temperature clinker calcination, typically consumes only one-third to one-half of its energy, and can substantially reduce CO_2_ emissions [8,9,10].

At present, extensive investigations have been conducted worldwide on precursor types and the optimization of alkali activators for geopolymer materials, and relevant research on the regulation of mechanical properties has become relatively mature [11,12,13,14]. Nevertheless, excessive shrinkage deformation, as an inherent drawback of geopolymers, severely limits their large-scale engineering applications. Experimental results demonstrate that under natural curing conditions, the shrinkage strain of geopolymers is generally more than four times that of OPC [15]. The shrinkage of geopolymers primarily consists of chemical shrinkage and drying shrinkage. Chemical shrinkage is mainly governed by the category and reaction activity of precursors [16,17], whereas drying shrinkage is closely associated with the pore structure characteristics of the matrix and the loss of pore solution inside the material.

Seyrek [18] and Hanumananaik [19] compared geopolymers with ordinary cement-based materials, and concluded that the high shrinkage of geopolymers can be mainly attributed to three factors: high capillary stress caused by mesopore water loss, formation of low-density C-A-S-H gels, and the absence of crystalline phases such as calcium hydroxide (Ca(OH)_2_) and ettringite (AFt) that can compensate for volume deformation [20]. To mitigate the shrinkage of geopolymers, Taghvayi et al. [21] experimentally found that with a constant alkali content, when the modulus of water glass increased from 0.45 to 1.05, the 400-day drying shrinkage of geopolymers rose by three times. Mastali et al. [22] reported that shrinkage strain increased with increasing contents of active SiO_2_ and Na_2_O in alkali activators; when the content increased from 1.0% to 1.5%, the 7-day autogenous shrinkage increased by a factor of 2.7. Reducing the alkali concentration and adjusting the silicate modulus can effectively reduce the shrinkage strain of geopolymers. Relevant studies have verified that regulating mixing proportions, optimizing alkali activators and modifying mineral admixtures can improve the gel structure and alleviate the shrinkage deformation of geopolymers [23,24]. Nevertheless, existing research on shrinkage regulation mainly focuses on conventional slag-based and fly ash-based geopolymers. Studies on the shrinkage characteristics and regulatory mechanisms of lithium slag-based geopolymers remain limited, failing to meet the requirements of practical engineering applications.

Fiber reinforcement is recognized as an effective means to improve the toughness and volume stability of geopolymers, and various fibers (inorganic, natural, metal, and synthetic) have been used in geopolymer shrinkage inhibition research [25,26,27]. Single-fiber reinforcement can exert bridging and stress-transfer effects to inhibit the development of microcracks and reduce shrinkage deformation.

Punurai et al. [28] found that basalt fiber can not only prolong the setting time and improve mechanical strength but also effectively restrain the drying shrinkage of geopolymers. Wang et al. [29] and Moradikhou [30] investigated the effect of the fiber volume fraction on the autogenous shrinkage performance of cement-based materials via shrinkage tests. The results indicated that the addition of PVA fiber reduces the autogenous shrinkage of cement-based materials. Li et al. [31] analyzed the failure mechanism of PVA fiber-reinforced geopolymers and revealed that the optimal fiber volume content is 2.0%. Excessive fiber addition causes fiber agglomeration and thus weakens the shrinkage inhibition effect. With the in-depth research on fiber-reinforced geopolymers, multi-scale synergistic modification with hybrid fibers has become an important technical approach to improving the volume stability and mechanical properties of materials. Tung et al. [32] demonstrated that combining different fiber types can achieve an excellent synergistic strengthening effect. Rational design of fiber types and mixing ratios enables geopolymers to meet diverse engineering requirements for strength, toughness and durability simultaneously.

With the widespread application of nanomaterials in material modification owing to their unique size effect, interfacial reinforcement effect and nano-filling effect, various nanomaterials have been adopted to optimize the properties of geopolymers. Among them, nano-silica, carbon nanotubes and graphene are the most commonly used modifiers. Zhong W.H. [33] conducted orthogonal tests and found that carbon nanotubes exhibit pronounced nano-filling and bridging effects, which can effectively prolong the setting time, greatly improve durability, and enhance the impermeability and freeze–thaw resistance of geopolymers. Arpitha B et al. [34] concluded that nano-alumina and graphene oxide facilitate the formation of C-A-S-H gels. This shortens the setting and hardening time of geopolymer mortar, increases mortar viscosity, and consequently reduces workability. Studies by Hu et al. [35] and Liu et al. [36] demonstrated that incorporating nano-silica into geopolymer systems can fully promote geopolymerization and optimize the matrix microstructure. Accordingly, the mechanical strength and rheological properties of geopolymers are remarkably enhanced, providing an effective strategy for high-performance modification.

Current fiber modification for geopolymers presents two key limitations. Most fibers degrade the paste’s workability and require a higher water–binder ratio, thereby impairing shrinkage control. In addition, existing studies mainly focus on millimeter-scale fibers, whereas the shrinkage regulation of nanofibers and the synergistic mechanism of hybrid macro-nanofibers in lithium slag-based geopolymers remain poorly understood [37,38]. Hooked-end steel fibers have negligible adverse effects on workability and are suitable for mitigating shrinkage, while carbon nanotubes can refine the pore structure and densify gel phases through nano-filling and interfacial reinforcement. The hybridization of the two fibers enables micro–macro structural optimization of lithium slag-based geopolymers [39].

Accordingly, this study systematically investigates the shrinkage characteristics and regulation mechanisms of lithium slag-based geopolymers to fill the above research gaps. The fiber–matrix interface provides the mechanical link between matrix shrinkage and fiber restraint: bond, friction, and hooked-end anchorage transfer axial stress through interfacial shear. Pull-out and shear-lag formulations provide a mechanics basis for this interpretation [40,41]. In sand-containing mortar, CNTs are additionally sensitive to agglomeration and to the limited volume of reactive gel around the relatively inert aggregate, so their local nano-scale action need not translate directly into a large specimen-scale shrinkage reduction [33,42,43].

This research aims to clarify the shrinkage regulation law of lithium slag-based geopolymers, develop a simplified mechanistic framework rather than a fully calibrated prediction model, and elucidate the shrinkage-inhibition effect and underlying mechanism of cross-scale fibers (hooked-end steel fibers and carbon nanotubes). This study compares single- and hybrid-fiber systems, links MIP and SEM observations with the shrinkage response, and evaluates whether the hybrid response departs from a simple additive benchmark. The results provide a basis for volume-stability design and high-value lithium slag utilization, while engineering-scale performance is outside the scope of this study.

## 2. Experiments

### 2.1. Raw Materials

**Figure 1:**
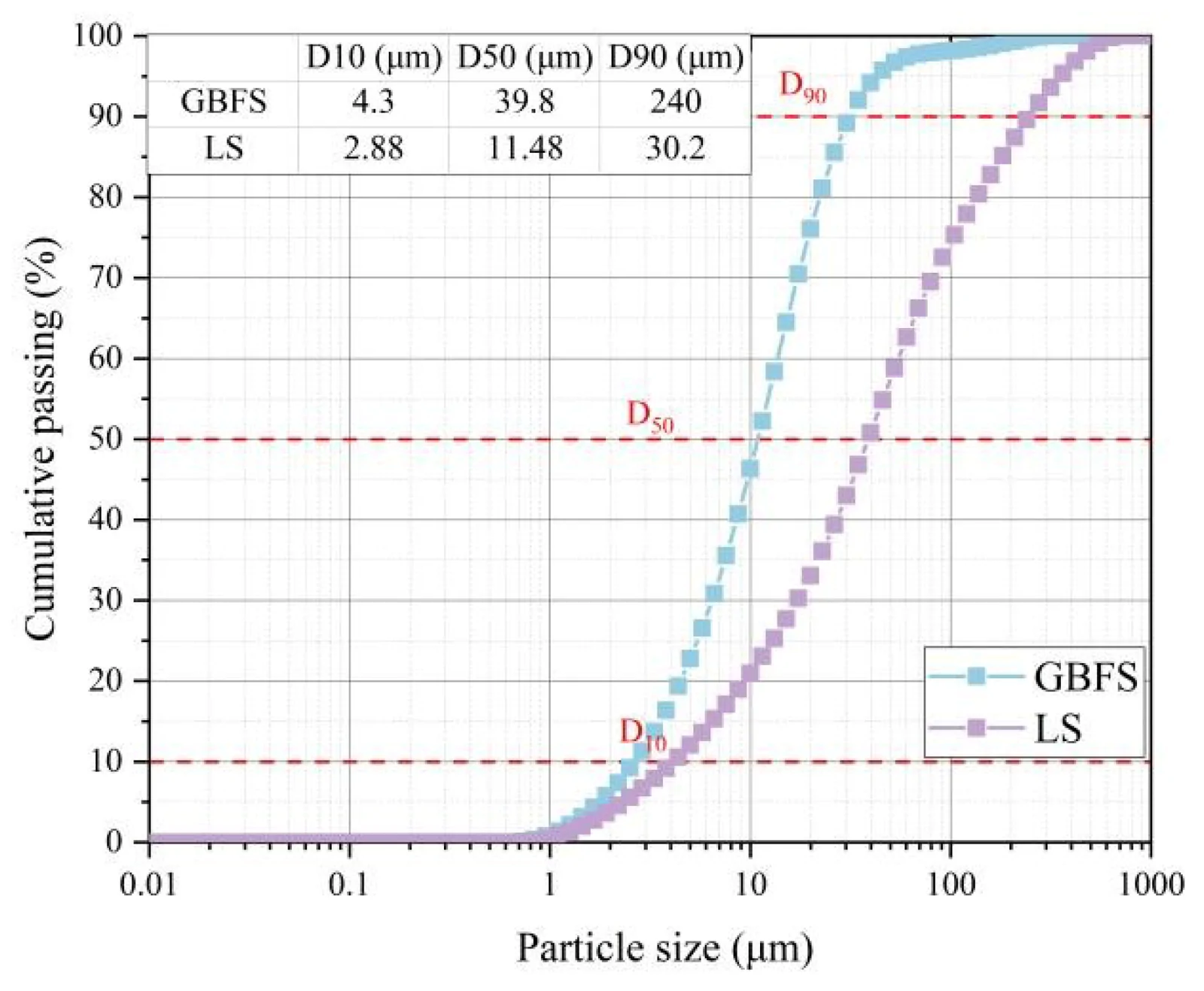
Raw material particle size of LS and GBFS [43].

In this study, industrial-waste lithium slag (LS) and granular blast-furnace slag (GBFS) were used as the primary alkali-activated precursors. LS was supplied by Yichun Ganfeng Lithium Industry Group Co., Ltd. (Xinyu, China), while GBFS was obtained from Henan Jiewei Environmental Protection Materials Co., Ltd. (Zhengzhou, China). The particle size distributions of the two precursors were tested via a laser particle size analyzer, and the corresponding results are illustrated in Figure 1. Flame atomic absorption spectroscopy (FAAS) and X-ray fluorescence spectroscopy (XRF) were employed to analyze the oxide compositions of LS and GBFS, with the detailed test data summarized in Table 1. Additionally, quartz sand with a particle size range of 80–120 mesh was used as the fine aggregate throughout the experiments.

**Figure 2:**
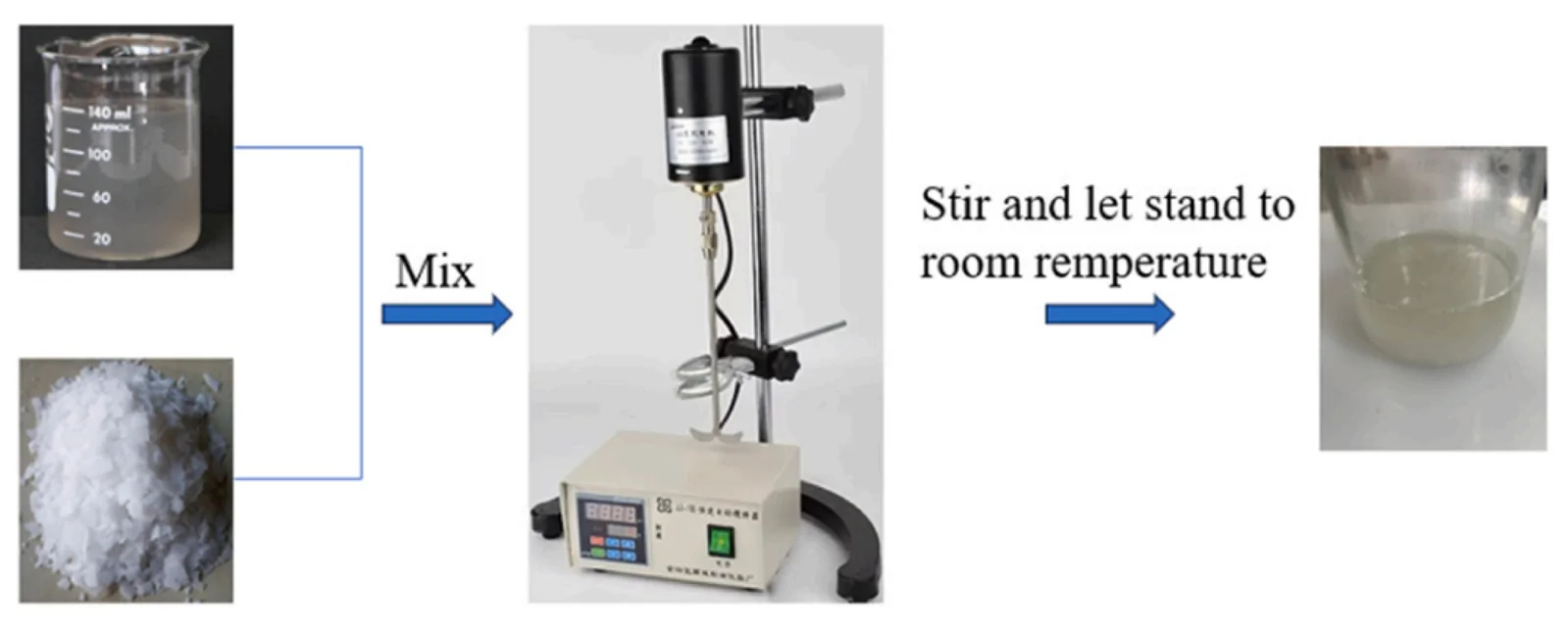
Preparation process of the dual-alkali activator.

In this experiment, the alkali activator was prepared from sodium silicate (Na_2_SiO_3_) and sodium hydroxide (NaOH), and its detailed preparation method is illustrated in Figure 2. The employed sodium silicate solution had mass fractions of sodium oxide (Na_2_O), silicon dioxide (SiO_2_) and water at 8.3%, 26.2% and 65.5%, respectively. The sodium hydroxide was a white granular solid with a purity of more than 99%. To regulate the reaction rate of alkali activation, NaOH was added to the sodium silicate solution to reduce the SiO_2_-to-Na_2_O molar ratio in the alkali activator, ultimately adjusting it to 1.2.

(1) $${\mathrm{Na}}_{2}{\mathrm{SiO}}_{3}+2\mathrm{NaOH}={\mathrm{Na}}_{2}O\cdot {\mathrm{SiO}}_{2}+{\mathrm{Na}}_{2}O+H_{2}O$$

**Figure 3:**
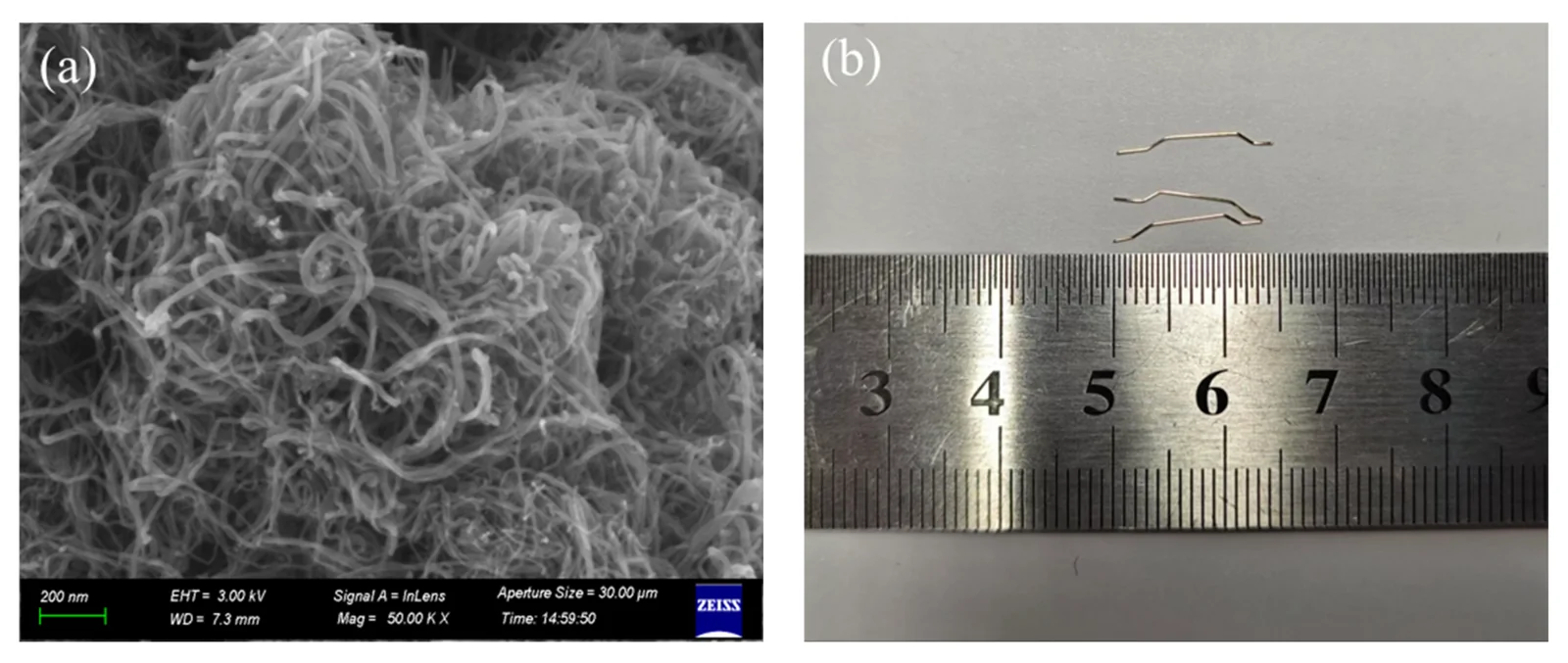
Images of (**a**) CNTs and (**b**) HSFs.

Carbon nanotubes (CNTs) exhibited excellent mechanical properties, along with small and uniformly distributed particle sizes, a large specific surface area, and good chemical stability, but poor dispersibility. The mass fraction of CNTs used in this study was 0~0.15% (Figure 3a), and the volume fraction of hooked-end steel fibers (HSFs) was 0~1.5% (Figure 3b). The performance parameters of both materials are detailed in Table 2. The CNTs employed in this experiment were purchased from Jiangsu Xianfeng Materials Technology Co., Ltd. (Taizhou, China), and the HSFs were from Hebei Kangsheng Materials Co., Ltd. (Hengshui, China). The CNTs were mixed with water (100 times the mass of the CNTs) and a sodium 1-dodecanesulfonate (SDS) dispersant with a mass fraction of 25%, and were then ultrasonically dispersed at 45 °C for 60 min to prepare a stable CNT suspension.

### 2.2. Preparation of Geopolymer Specimens

Table 3 lists the mixture proportions of the mortars prepared in this study. For all mixtures, the binder-to-sand ratio was fixed at 0.33, the alkali modulus and alkali equivalent of the activator were 1.2 and 7%, respectively, and the water-to-binder ratio of the geopolymer cementitious system was 0.37. Sixteen mortar groups were prepared to investigate the effects of CNT and HSF content. In the specimen labels, C denotes CNT content (0.05–0.15%) and H denotes the HSF volume fraction (0.5–1.5%). The selected CNT range (0.05–0.15 wt% of binder) and HSF range (0.5–1.5 vol%) were chosen as low-to-high dosage levels that cover the range used for nano-modified and fiber-reinforced geopolymer mortars in the cited literature [28,33,37,38]. The upper levels were kept below the dosage range at which agglomeration, entrapped air, or workability loss could obscure the reinforcement effect; the present study therefore treats the selected levels as an investigated range rather than an optimized universal dosage.

**Figure 4:**
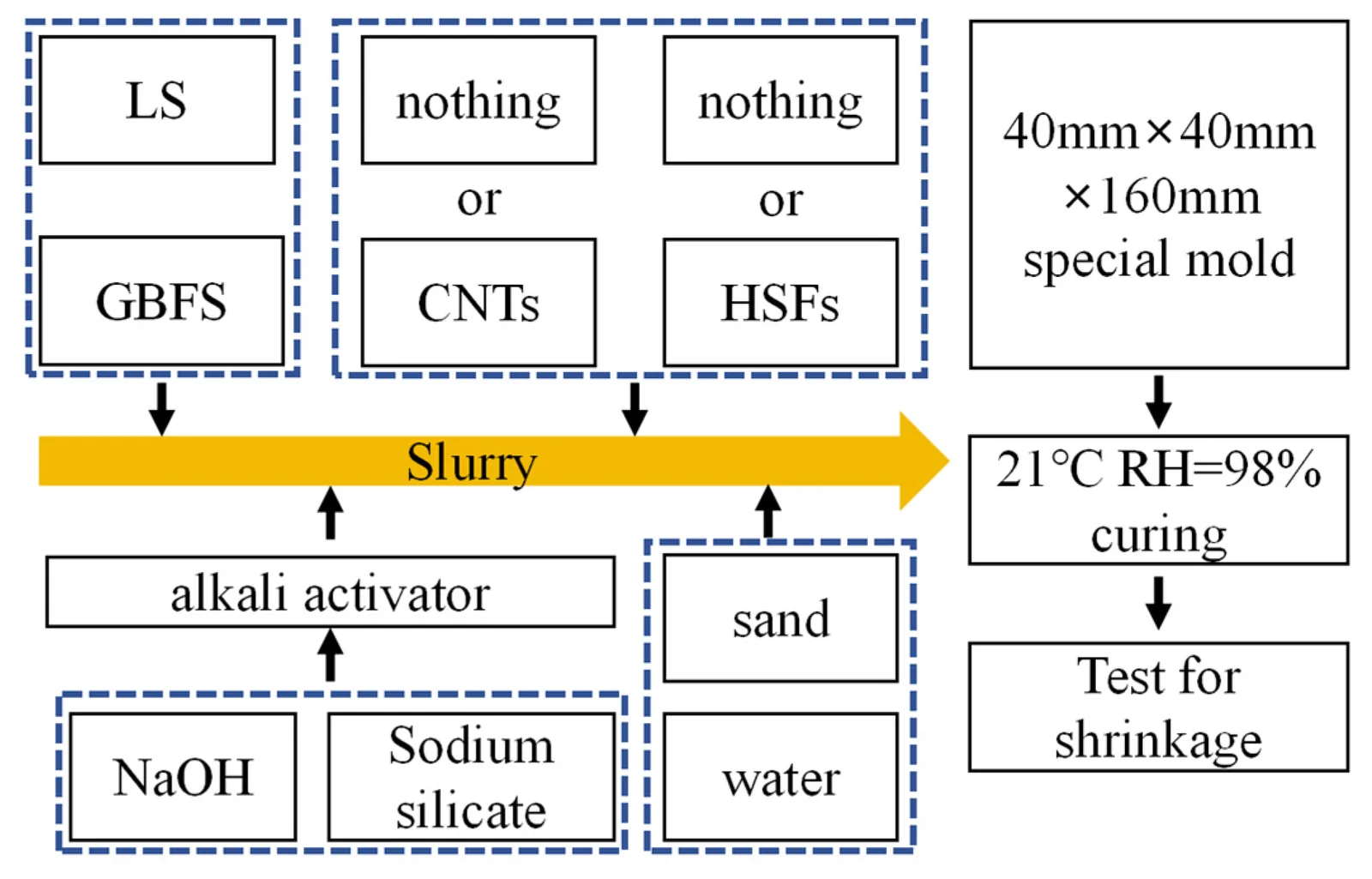
Critical fabrication steps for LSG specimens.

As shown in Figure 4, the preparation process of the geopolymer mortar is as follows: First, GBFS and LS were added into a mixer for dry mixing for 30 s, followed by the addition of the alkali activator with low-speed stirring for 120 s. Subsequently, the carbon nanotube suspension and HSFs were added in accordance with the mix proportions and stirred for 1 min, after which the remaining water and quartz sand were added sequentially with continuous stirring for another 1 min. Once all raw materials were uniformly mixed, the fresh geopolymer mortar was promptly poured into dedicated 40 mm × 40 mm × 160 mm molds. Prior to pouring, the inner walls of the molds were evenly coated with a mold release agent to facilitate demolding. The molds were then placed on a vibrating table and vibrated to compaction for 1 min, and vibration was ceased when tiny air bubbles continuously precipitated on the specimen surface; the surface of the test blocks was troweled flat with a scraper thereafter. Finally, the test blocks were covered with plastic wrap and placed in a constant-temperature, constant-humidity curing chamber for initial curing (temperature: 21 °C; relative humidity: 98%). The test blocks were demolded after 24 h and then returned to the curing chamber to reach the prescribed age under identical temperature and humidity conditions.

### 2.3. Test Methodologies

#### 2.3.1. Shrinkage Test

**Figure 5:**
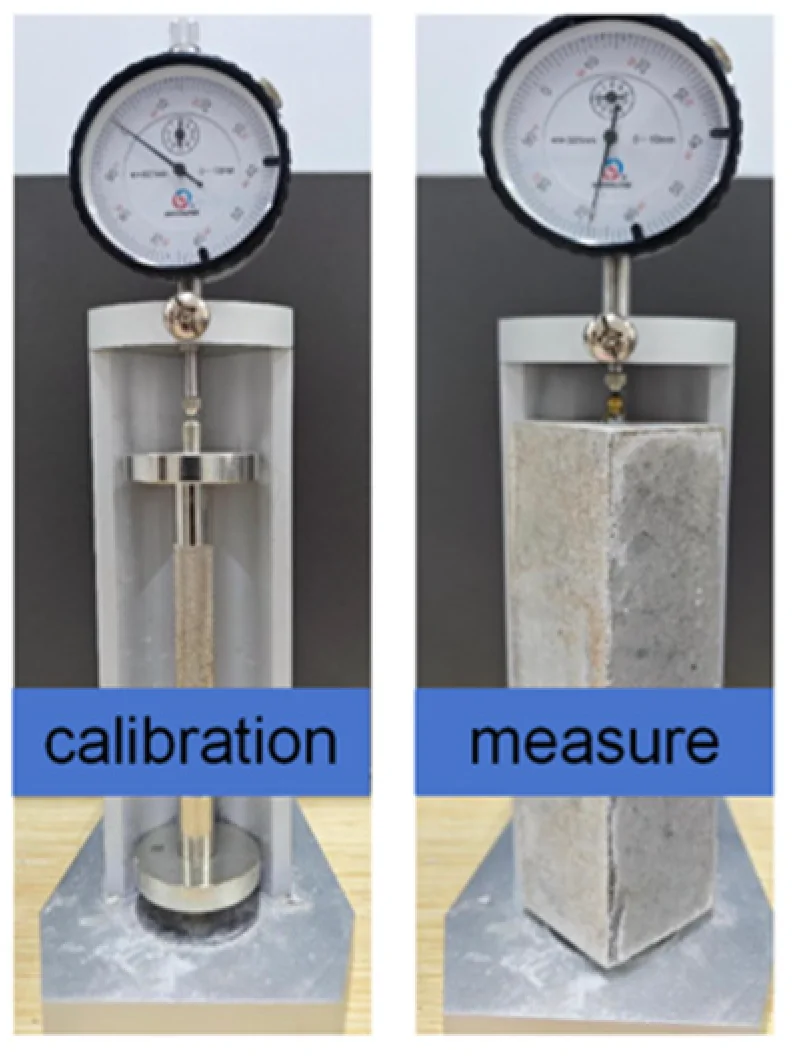
Shrinkage experimental facility [43].

To continuously characterize the shrinkage strain of the LSG during curing, length measurements were conducted in accordance with JGJT70-2009 [44] using a length comparator. An Ames dial gauge with a precision of 0.001 mm served as the testing apparatus. Each group contained three specimens, and average values were adopted for analysis. The initial length (*L*_0_) was measured right after demolding (Figure 5), followed by continuous curing in the chamber. *L_d_* is the length of the two copper nails of the sample. Specimen length (*L_t_*) was recorded on each day t since demolding. The shrinkage rate was calculated using the formula below. The shrinkage rate in Equation (2) was obtained from the change in epsilon over the corresponding time interval. The test data presented in the subsequent text report strain rather than strain rate. The specimens measured 40 mm × 40 mm × 160 mm. Each group contained three specimens (*n* = 3), and the plotted symbols denote mean values with error bars representing ± standard deviation.

(2) $$\varepsilon_{t}=\frac{L_{0}-L_{t}}{L-L_{d}}$$

#### 2.3.2. Microstructure Analysis of Geopolymer

MIP sample preparation and method: Samples were taken from the central part of the same specimen that had completed the 90-day shrinkage rate test. The sample mass was approximately 1 g, with a size of 3 mm × 3 mm × 3 mm. The vacuum freeze-drying method (Spanish Telstar-85 plus, Azbil Telstar Technologies, S.L.U., Terrassa, Spain) was used. The drying conditions were −50 °C, vacuum degree of 10 Pa, and drying time of 24 h, until the sample mass change was less than 0.1%. The mercury intrusion pore method (MIP) experiment was performed on a US Micromeritics AutoPore 9600 instrument (Micromeritics Instrument Corporation, Norcross, GA, USA) at 0–30,000 psi (0–206.84 MPa) and measured the porosity and pore size of the sample.

In addition, the data in the MIP experiment were calculated by the space-filling model [45] to obtain the fractal dimension of the sample. The calculation method is as follows:

(3) lgV=lgC+(3−D)lgφ

where *V* is the pore volume or mercury intake, *φ* is the aperture, and *C* is a constant.

Washburn equation:

(4) $$d=\frac{4\gamma \cos\theta }{P}$$

The MIP test parameter setting has the contact angle (*θ*) set at 140°. The surface tension of mercury (*γ*) at 25 °C is 480 mN/m.

A field-emission scanning electron microscope (FE-SEM, ZEISS Sigma 360, Zeiss, Oberkochen, Germany) was employed to characterize the microstructure and morphology of the LSG specimens following the 90-day shrinkage test. SEM images were utilized to observe fiber dispersion, interfacial features, cracks, and distinguishable pore characteristics. No XRD, FTIR, or TGA measurements were conducted for the present batch. Accordingly, references to C-A-S-H or other reaction products below are literature-based interpretations, and are not presented as phases directly identified by this study.

## 3. Results and Analysis

### 3.1. Dry Shrinkage Strain

**Figure 6:**
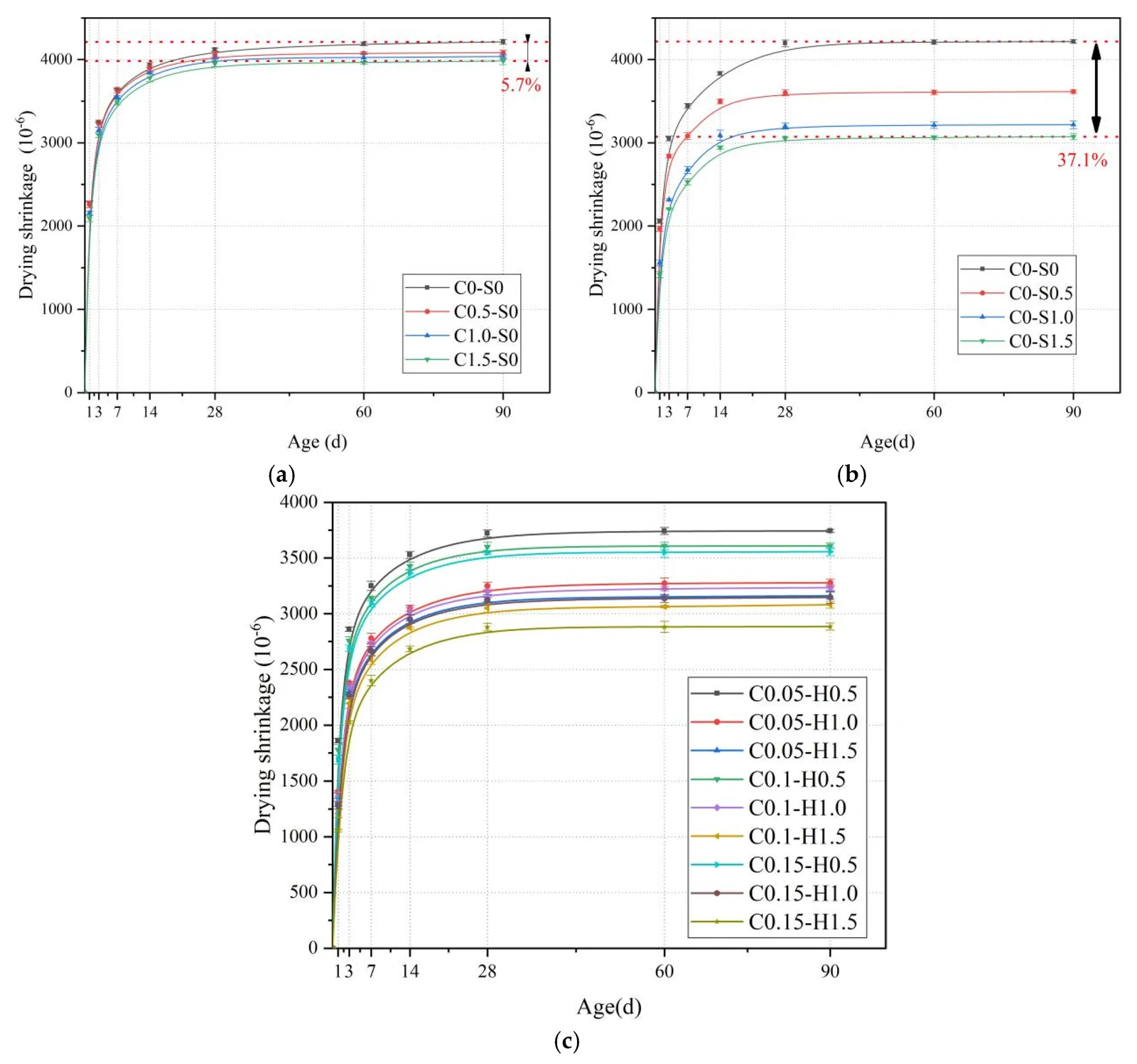
Microstrain of drying shrinkage under different fiber ratios (**a**) CNTs group; (**b**) HSF group; (**c**) hybrid fiber group.

Figure 6a presents the variation in shrinkage microstrain with age for LSG at different levels of CNT content. It can be clearly observed from this figure that all groups exhibit the characteristic of a fast shrinkage rate in the early stage (1–14 d) and stable shrinkage after 28 d; moreover, the shrinkage degree of LSG shows a slight decreasing trend with the increase in the CNT mass fraction. For the C0.15-H0 group, where the CNT mass fraction was 0.15%, the shrinkage value at 90 days was 3983 με. This value is 5.7% lower than that of the C0-H0 control group, which contained no CNTs. These results indicate that CNTs can inhibit the shrinkage of LSG to a certain extent, mainly due to the nano-filling and bridging effects of CNTs, which can refine pores and alleviate volume shrinkage caused by water evaporation. This interpretation is consistent with reported nano-filling, crack-bridging, and dispersion-dependent effects of CNTs in cementitious and geopolymer composites [33,41,42].

The shrinkage-inhibiting effect of CNTs, however, was limited in the LSG mortar system. CNTs are most effective within nanoscale gel products, where they can fill pores, bridge microcracks, and improve local structural compactness. In LSG mortar, a large volume of inert and relatively non-deformable quartz sand occupies the matrix, while the proportion of nanoscale gel products is comparatively limited. As a result, the action range of CNTs is mainly confined to the gel phase and cannot provide uniform restraint throughout the entire specimen, which explains the modest reduction in macroscopic shrinkage observed. This scale dependence is also consistent with recent observations that pore refinement and shrinkage reduction in fiber-reinforced geopolymers do not necessarily vary in direct proportion because fiber restraint and matrix pore structure act through different mechanisms [46].

From Figure 6b, it can be observed that with the gradual increase in V_H_, the shrinkage value of LSG shows a significant decreasing trend. When *V*_H_ reaches 1.5%, the dry shrinkage value of LSG decreases to 3706 με (90 days), which is 26.8% lower than that when *V*_H_ is 0%. These data fully show that HSFs have a very obvious inhibitory effect on the shrinkage of LSG. The skeleton effect of HSFs can build a stable support structure in the mortar system. In the drying process, when the free water continues to evaporate, the capillary stress leading to shrinkage will be generated, and the skeleton structure formed by HSFs can effectively resist this stress, thus significantly reducing the dry shrinkage value of the LSG and playing a key role in improving the stability of LSG in a dry environment.

**Figure 7:**
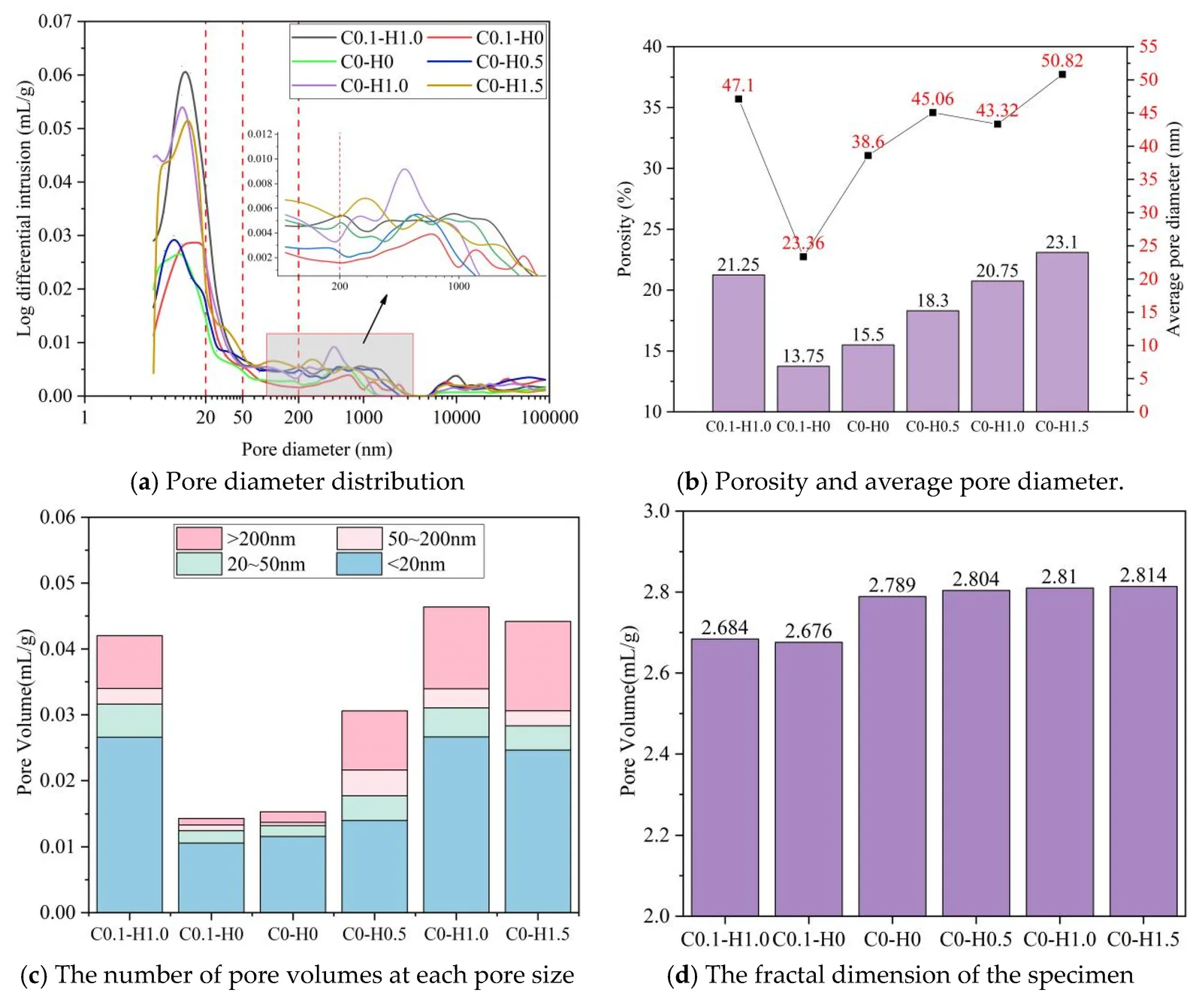
Pore characteristics of LSG with different fibers.

Figure 7c illustrates the effect of hybrid fibers with different dosages on the shrinkage strain of LSG, while Table 4 further reveals the synergistic shrinkage inhibition effect of multi-scale fibers by quantifying the discrepancy between experimental and theoretical values. In this table, the theoretical shrinkage reduction rate C+H is obtained by summing the experimental reduction rates of CNT-only and HSF-only specimens, whereas the experimental shrinkage reduction rate C+S is directly measured from multi-scale hybrid fiber specimens.

It is evident from the data in Table 4 that the drying shrinkage reduction rate of multi-scale fiber mortars is significantly higher than that of mortars containing only CNTs or HSFs at the same dosage. For instance, in the C0.15-H1.5 group, the shrinkage reduction rate of CNT-only specimens C_exp_ is 5.82%, and that of HSF-only specimens H_exp_ is 23.70%, yielding a theoretical cumulative reduction rate of 29.52%, whereas the measured reduction rate of the hybrid fiber specimen reaches 31.33%, representing an increase of 1.81 percentage points over the theoretical value. In the C0.1-H1.0 group, the theoretical value is 23.08%, while the experimental value is 23.70%, exceeding the theoretical value by 0.62 percentage points. Even in the low-dosage C0.05-H0.5 group, the experimental value (11.30%) is slightly higher than the theoretical value (11.16%).

By comparing all groups, it can be observed that the experimental drying shrinkage reduction rates (C+H)_exp_ of multi-scale hybrid fibers are consistently higher than the theoretical cumulative values (C+H)_cal_. Moreover, this discrepancy increases with increasing CNT and HSF dosages, rising from 0.14% in the C0.05-H0.5 group to 1.81% in the C0.15-H1.5 group. This result confirms that at the same fiber dosage, multi-scale hybrid fibers exhibit a significant synergistic effect, and their drying shrinkage inhibition performance is notably superior to the additive effect of individual fibers. The experimental hybrid reduction rates were not consistently higher than the theoretical additive values: the deviations ranged from −0.57 to +1.81 percentage points across the mixtures listed in Table 4. The largest positive deviation occurred for C0.15-H1.5, but this difference is small relative to the experimental scatter and was not tested with a formal significance test. The results therefore suggest a possible dosage-dependent positive interaction, rather than establishing a universal or statistically significant synergistic effect. The complementary functions of CNT nano-scale pore refinement and HSF macro-scale restraint remain a plausible mechanism for the observed deviation.

### 3.2. Pore Structure

Figure 7 shows that different fibers changed the pore structure of LSG in distinct ways. For the mixtures containing only HSFs, the pore size distribution shifted from a narrow peak dominated by small pores (<20 nm) in the control mixture to a multi-peak pattern with additional pores appearing in the mesopore (20–50 nm) and macropore (>50 nm) ranges. As the HSF dosage increased from 0 to 1.5%, the total porosity increased from 15.5% to 23.1%, and the average pore diameter increased from 23.36 to 50.82 nm. The macropore volume also increased, and the fractal dimension rose from 2.676 to 2.814, indicating a rougher and more irregular pore network. This is mainly attributed to the geometric mismatch between steel fibers and the geopolymer matrix, weak interfacial transition zones, local microcracks, and air entrapped during fiber incorporation. Thus, HSFs tend to loosen the local pore structure around the fiber–matrix interface, even though they can improve the macroscopic dimensional stability of the composite.

In contrast, CNTs refined the pore structure. Compared with the control mixture, C0.1-H0 showed a pore size distribution more concentrated below 20 nm, without obvious macropore peaks. Its porosity decreased to 13.75%, about 11% lower than that of C0-H0, while the average pore diameter did not increase significantly. This low macropore volume and slightly reduced fractal dimension indicate that CNTs made the pore network more compact and regular. This refinement can be explained by the nano-filling and microcrack-bridging effects of CNTs: well-dispersed CNTs can fill nanoscale defects, connect adjacent gel products, and inhibit the growth of larger pores. However, its refinement effect is mainly limited to the gel phase. Because the mortar contains a large amount of inert quartz sand, the volume of reactive gel available for CNT modification is restricted, which helps explain why the shrinkage reduction caused by CNTs remained moderate.

The hybrid system showed competition between these two mechanisms. The C0.1-H1.0 mixture had a porosity of 21.25%, slightly higher than that of the HSF-only mixture with the same HSF dosage, suggesting that HSF-related interfacial voids and possible air entrainment still dominated the total pore volume. However, CNTs partly refined the interfacial micropore network, as reflected by the lower fractal dimension of C0.1-H1.0 (2.804) compared with C0-H1.0 (2.810). Therefore, the CNTs did not completely offset the porosity increase caused by the HSFs, but they improved the compactness of local weak regions near the interface. The SDS used in the CNT suspension may have also influenced porosity. Surfactant-assisted sonication improves CNT dispersion, but SDS may stabilize bubbles during mixing and therefore contribute to additional pores. Because no SDS-only control group was designed, its independent contribution cannot be quantified here and is only discussed as a plausible factor [40,46].

The comparison between the MIP results and shrinkage further indicates that total porosity alone cannot predict the macroscopic shrinkage of fiber-reinforced LSG. For CNT-only mixtures, the decrease in porosity from 15.5% to 13.75% is consistent with pore refinement and a modest shrinkage reduction of 4.15%. This suggests that CNTs mainly reduce capillary-stress-related deformation by densifying the gel phase and bridging small cracks. However, for HSF-containing mixtures, porosity increased from 15.5% to 23.1%, and the average pore diameter increased from 23.36 to 50.82 nm, while the 90-day shrinkage decreased by 26.8%. This counterintuitive result shows that the shrinkage behavior of HSF-reinforced LSG is governed not only by pore structure but also by mechanical restraint. Owing to their high elastic modulus, interfacial adhesion, frictional resistance, and hooked-end anchorage, HSFs can transfer tensile and shear stresses at the fiber–matrix interface and restrain matrix contraction during drying. Once matrix shrinkage induces relative displacement, these interfacial actions consume deformation energy and delay crack opening. Therefore, in CNT-dominated mixtures, pore refinement is the main shrinkage-reduction mechanism; in HSF-dominated mixtures, fiber-induced mechanical restraint can outweigh the adverse effect of increased porosity. In the hybrid system, CNTs improve local pore compactness, while HSFs provide a broader restraining framework. These results demonstrate that MIP parameters should be interpreted together with fiber–matrix interfacial behavior when evaluating the shrinkage mechanism of LSG].

### 3.3. Microstructure

**Figure 8:**
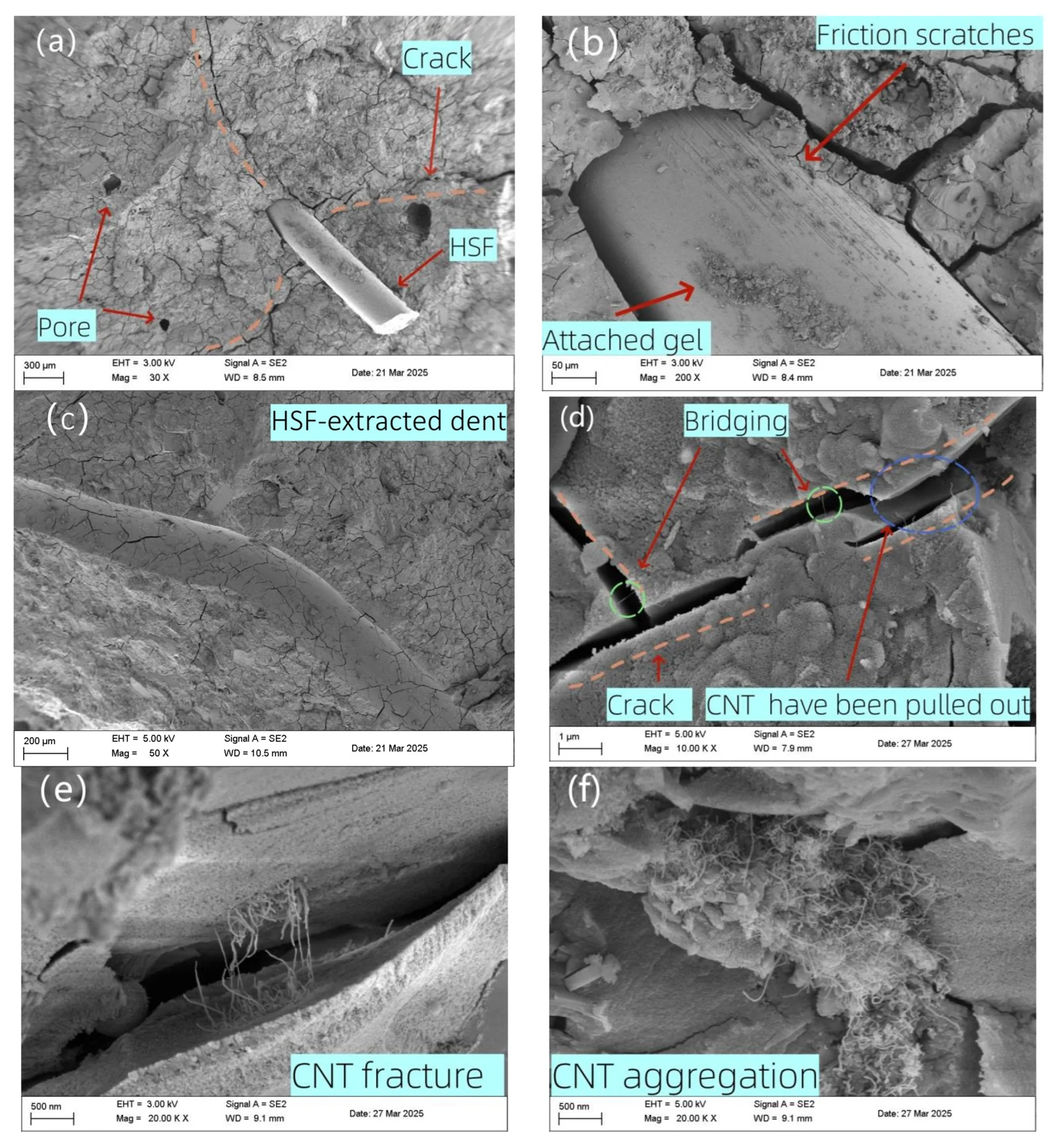
Characterization of HSFs and CNTs in LSG under microcosmic conditions (**a**) Distribution of HSFs in the matrix; (**b**) Friction scratches generated during HSF pull-out; (**c**) Residual dent after HSF pull-out; (**d**) Crack-bridging effect of CNTs; (**e**) Crack-bridging effect of CNTs; (**f**) Entanglement of CNTs.

As shown in Figure 8a–d, HSFs showed no obvious deformation after specimen failure. In most cases, one end of the HSF remained embedded in the matrix while the other end was pulled out, with hydration or geopolymerization products adhering to the exposed surface. Clear grooves and pull-out traces were observed in the matrix, indicating good interfacial bonding between the steel fiber and the matrix. Wide cracks around the fibers revealed local stress concentration. Scratches and attached gel products on the HSF surface further indicated that frictional resistance developed during matrix deformation. When matrix shrinkage induces relative displacement, the fiber strain remains small, and interfacial friction restrains deformation. Once the local stress exceeds the interfacial resistance, slip occurs, and cracks develop around the fiber.

Figure 8d,e show CNTs bridging and being pulled out near cracks adjacent to fibers. In cracks approximately 1 µm wide, CNTs were stretched but remained intact; in cracks approximately 2 µm wide, CNTs were pulled out and curled. This behavior indicates that CNTs primarily inhibit the propagation of microcracks with widths below approximately 2 µm via a bridging mechanism. The effectiveness of CNTs is also strongly dependent on their dispersion state. As shown in Figure 8f, CNT agglomeration reduces their crack-bridging efficiency.

Compared with CNTs, HSFs are less prone to agglomeration or entanglement, and individual steel fibers can more reliably exert a bridging and restraining effect. CNTs are most effective during the early stage of microcrack development, but their influence weakens as deformation localizes and cracks widen. Therefore, HSFs provide a broader and stronger range of deformation inhibition in LSG. At the same time, CNTs can suppress microcracks induced by stress concentration around HSFs, enabling multi-scale synergistic regulation from the millimeter scale to the micrometer scale.

### 3.4. Shrinkage Inhibition Mechanism

**Figure 9:**
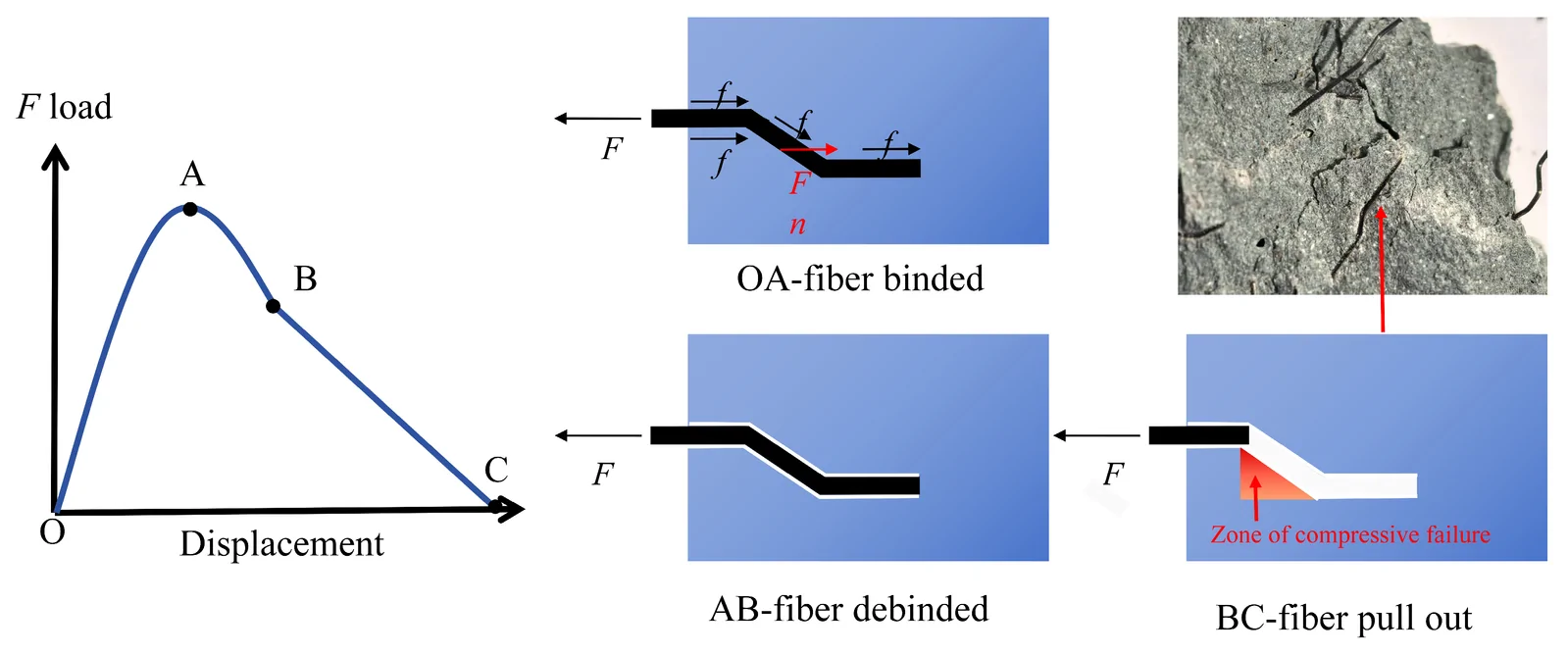
The stress-displacement curve of the HSFs extracted from LSG and the stage failure mechanism. This arrow indicates the corresponding stage of fiber pull out in this image.

The shrinkage of LSG consists of two components: autogenous shrinkage and drying shrinkage. During shrinkage, the matrix shrinkage strain differs from that of the fibers, and interfacial adhesion between the matrix and fibers resists matrix shrinkage and interfacial slip. As shown in Figure 9, the pull-out process of HSFs from the matrix can be roughly divided into three stages: OA, AB, and BC. In the OA stage, fully bonded, the fibers remain completely bonded to the matrix and hooked-end fibers resist deformation-induced stress through a combination of bonding friction and bearing force; in the AB stage, partially debonded, the fibers are partially bonded, the friction between the matrix and fibers exceeds the bonding force, and the interface begins to fail; and in the BC stage, fiber pull-out, the bonding friction between the fibers and matrix is nearly exhausted, deformation is primarily resisted by the bearing force on the inner side of the hooked ends, and after the matrix on the inner side of the hooked ends undergoes compressive failure, the fibers begin to slip and are eventually pulled out. Since the strain level of LSG shrinkage is far lower than the critical strain that triggers interfacial debonding in pull-out tests, matrix shrinkage only causes minor absolute displacement of fibers, which is insufficient to induce fiber debonding or pull-out, indicating that the ability of HSFs to resist matrix shrinkage is fully manifested in the OA stage, where interfacial friction and bonding dominate, without relying on subsequent debonding or pull-out mechanisms. This indicates that HSFs resist matrix contraction primarily through interfacial friction and adhesion, and that the stress conditions are similar to those in the OA stage. The OA-stage interpretation is consistent with the small deformation regime of interfacial shear-lag models [46] and with reported shrinkage restraint by hooked-end steel fibers [40].

**Figure 10:**
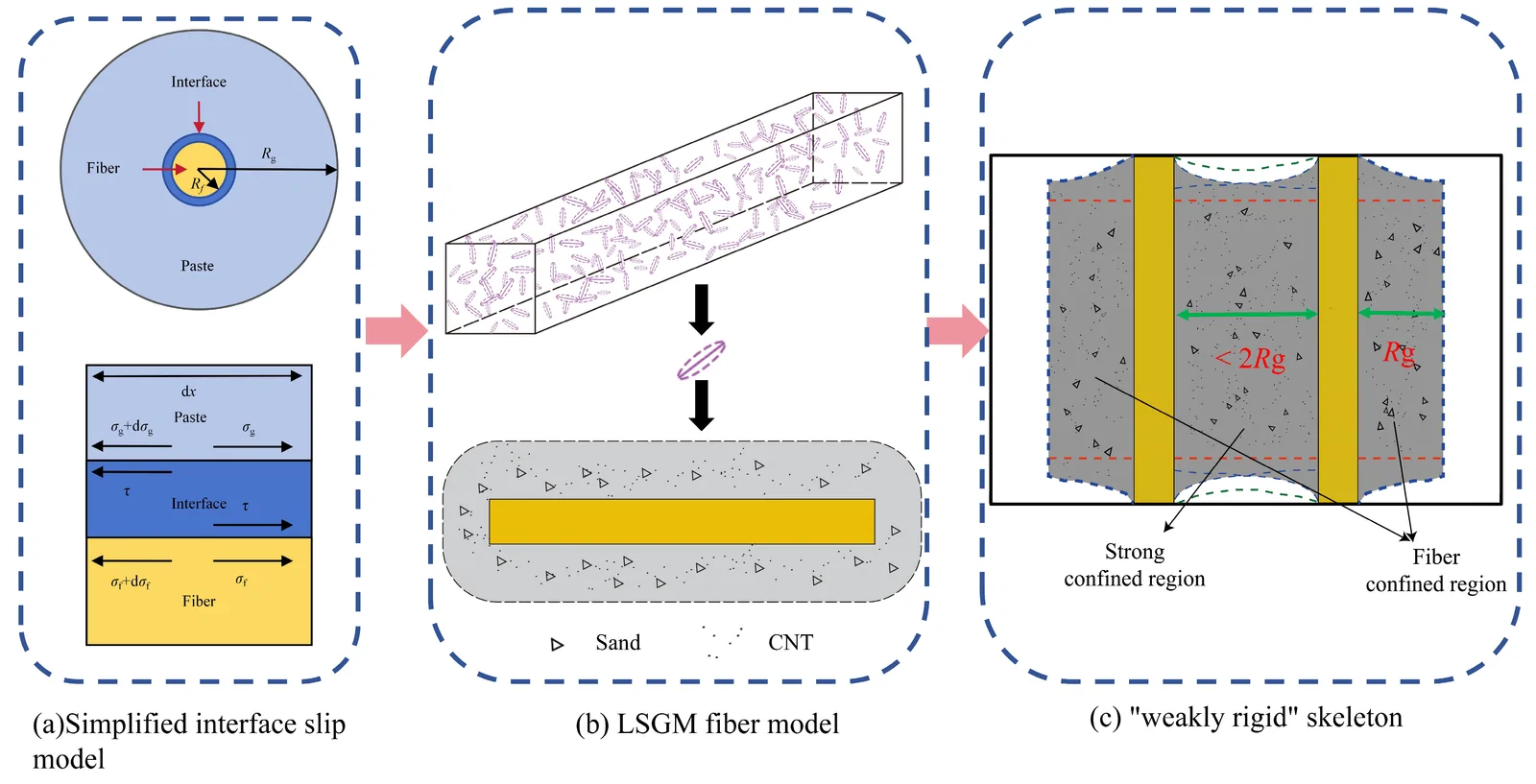
The formation mechanism of the fiber-restrained zone based on interface shear transfer and the weakly rigid framework.

The interface friction and adhesion force are uniformly simplified as the interface shear stress. Assuming that there is only shear stress at the fiber–LSG interface and that it is uniformly distributed along the interface’s thickness direction, the simplified interface-bonding slip model is shown in Figure 10a. The equivalent radii of the fiber and the LSG matrix are represented by *R_f_* and *R_g_*, respectively. The obtained stress and displacement distributions in the matrix, fiber, and interface are shown in Figure 10. Based on the above assumptions, the mechanical equation of the interface bond-slip model are presented in Formulas (5)–(8): where is the shear stress at the interface, and σ*_f_* and σ*_g_* refer to the stress along the fiber axis in the fiber and the matrix, respectively. Formula (5) represents the overall axial force balance formula, Formula (6) is the stress gradient formula for the fiber elemental segment, Formula (7) is the stress gradient formula for the matrix elemental segment, and Formula (8) is the geometric dimension correlation formula. Formulas (5)–(8) are used here as a simplified shear-lag representation of stress transfer. Because the present study did not independently measure the full interfacial bond-slip law, fiber spacing, or local shear-stress field, the equations are not calibrated as a quantitative predictive model.

(5) $$\sigma_{f}{R_{f}}^{2}+\sigma_{g}{R_{g}}^{2}=0$$

(6) $$\frac{d\sigma_{g}}{dx}=\frac{2\tau }{R_{f}}$$

(7) $$\frac{d\sigma_{g}}{dx}=-\frac{2\tau R_{f}}{R_{g}}$$

(8) $${R_{g}}^{2}=-\frac{{R_{f}}^{2}\sigma_{f}}{\sigma_{g}}$$

Based on the axial force equilibrium and stress gradient relationships described in Formulas (5)–(8), it is evident that the extent to which fibers can suppress matrix shrinkage is restricted to a finite range. The matrix region centered on a fiber with a radius of *R_g_* is hereby defined as the fiber-confined zone. As illustrated in Figure 10b, fibers are randomly dispersed within the matrix and form these fiber-confined zones via interfacial shear stress, thereby resisting shrinkage over a localized range. However, when the inter-fiber spacing is less than 2*R_g_*, the confined zones of adjacent fibers spatially overlap: the matrix in the overlapping region is simultaneously subjected to shear stress constraints from fibers on both sides, forming a strongly confined region with stress superposition (Figure 10b). This bidirectional confinement not only significantly enhances local shrinkage resistance but also enables mechanical coupling between discrete fibers through stress transfer. Ultimately, fibers are interconnected with their respective fiber-confined zones via these strongly confined regions, constructing a three-dimensional weakly rigid skeleton. This skeleton is essentially a synergistic load-bearing network formed by the interconnection of discrete fibers through confined zones; it extends the localized shrinkage-inhibiting effect to the entire matrix, thereby effectively suppressing macroscopic shrinkage deformation. This mechanism also provides a clear physical picture for understanding the shrinkage resistance of fiber-reinforced composites.

## 4. Conclusions

In the study of LSG, the effects of different mix ratios and multi-scale fiber content levels on its mineral composition, pore structure, and drying shrinkage characteristics were analyzed. Through experimental exploration and data analysis, the following main conclusions are drawn:

(1) Both CNTs and HSFs reduced the drying shrinkage of LSG, but their magnitudes and mechanisms differed. CNTs mainly produced a limited reduction through nano-filling and microcrack bridging; the 90-day shrinkage strain of C0.15-H0 decreased by 5.7% compared with the control. HSFs provided stronger macroscopic restraint, and C0-H1.5 decreased by 26.8%. The C0.15-H1.5 hybrid mixture achieved the largest measured reduction: 31.33%.

(2) The two fibers regulated the pore structure and microstructure of LSG through different mechanisms. CNTs refined the pore structure, reduced porosity, and inhibited the development of microcracks, but their effect was limited by dispersion quality and the relatively small proportion of nanoscale gel products in the mortar. HSFs slightly increased porosity because of interfacial pores and weak transition zones, but they bonded well with the matrix and effectively restrained deformation through friction, adhesion, and hooked-end anchorage. These mechanisms act at different scales and should not be inferred from porosity alone.

(3) Interfacial shear transfer provides a plausible explanation for fiber-confined zones. When adjacent zones overlap, they can form a weakly rigid framework that transfers local restraint to the surrounding matrix. The hybrid results show a dosage-dependent deviation from the additive benchmark, but the available n = 3 data do not establish statistical significance; the mechanism should therefore be regarded as a supported conceptual framework.

The present study did not include XRD, FTIR, or TGA characterization, an SDS-only control, or direct measurements of the complete fiber–matrix bond-slip law and fiber spacing. The fiber-confined-zone model is consequently presented as a mechanistic interpretation rather than a quantitatively validated prediction model. Future work should combine phase analysis, dispersant control, direct pull-out parameters, and larger replicate numbers to test the statistical significance of the small deviation from additivity.

## Figures and Tables

**Table 1:** Raw material component properties [43].

Chemical Compositions (%)	SiO_2_	CaO	Al_2_O_3_	Fe_2_O_3_	MgO	SO_3_	Li_2_O	Others
GBFS	34.2	34	17.6	1.01	6.21	1.62	/	5.36
LS	39.8	16.9	15.3	3.98	1.40	9.74	0.26	12.62

**Table 2:** Size parameters of CNTs and HSFs.

Mixture	Length (mm)	Diameter (um)	Elasticity Modulus (GPa)
CNTs	(1–3) × 10^−2^	(1–3) × 10^−3^	130
HSFs	15	75	210

**Table 3:** The mix ratio of the LSG sample.

Samples	Modulus (M)	Base Equivalent (S)	Water–Solid Ratio (W)	Rubber–Sand Ratio (C)	CNTs (%)	HSF (%)
C0-H0	1.2	7%	0.37	0.33	0%	0%
C0.05-H0	1.2	7%	0.37	0.33	0.05%	0%
C0.1-H0	1.2	7%	0.37	0.33	0.10%	0%
C0.15-H0	1.2	7%	0.37	0.33	0.15%	0%
C0-H0.5	1.2	7%	0.37	0.33	0%	0.50%
C0-H1.0	1.2	7%	0.37	0.33	0%	1%
C0-H1.5	1.2	7%	0.37	0.33	0%	1.50%
C0.05-H0.5	1.2	7%	0.37	0.33	0.05%	0.50%
C0.05-H1.0	1.2	7%	0.37	0.33	0.05%	1%
C0.05-H1.5	1.2	7%	0.37	0.33	0.05%	1.50%
C0.1-H0.5	1.2	7%	0.37	0.33	0.10%	0.50%
C0.1-H1.0	1.2	7%	0.37	0.33	0.10%	1%
C0.1-H1.5	1.2	7%	0.37	0.33	0.10%	1.50%
C0.15-H0.5	1.2	7%	0.37	0.33	0.15%	0.50%
C0.15-H1.0	1.2	7%	0.37	0.33	0.15%	1%
C0.15-H1.5	1.2	7%	0.37	0.33	0.15%	1.50%

**Table 4:** Experimental and theoretical values of drying shrinkage reduction rates of mono-doped and mixed fibers.

Group	Drying Shrinkage Decline Rate			
	C_exp_	S_exp_	(C+H)_exp_	(C+H)_cal_
C0.05-H0.5	2.24%	8.92%	11.30%	11.16%
C0.05-H1.0	2.24%	18.93%	22.51%	21.17%
C0.05-H1.5	2.24%	23.70%	25.13%	25.94%
C0.1-H0.5	4.15%	8.92%	14.16%	13.07%
C0.1-H1.0	4.15%	18.93%	23.70%	23.08%
C0.1-H1.5	4.15%	23.70%	27.28%	27.85%
C0.15-H0.5	5.82%	8.92%	15.36%	14.74%
C0.15-H1.0	5.82%	18.93%	25.61%	24.75%
C0.15-H1.5	5.82%	23.70%	31.33%	29.52%

## Data Availability

The original contributions presented in this study are included in the article. Further inquiries can be directed to the corresponding author.
